# Challenges associated with tracking resources allocation for reproductive health in sub-Saharan African countries: the UNFPA/NIDI resource flows project experience

**DOI:** 10.1186/s12978-015-0033-8

**Published:** 2015-05-05

**Authors:** Estelle M Sidze, Erik Beekink, Beatrice W Maina

**Affiliations:** African Population and Health Research Center (APHRC) APHRC Campus, Manga Close Off Kirawa road, P.O. Box 10787-00100, Nairobi, Kenya; UNFPA Resource Flows Project, Netherlands Interdisciplinary Demographic Institute (NIDI), P.O. Box 11650, NL-2502 AR The Hague, The Netherlands

**Keywords:** Reproductive health, Resource tracking, Sub-Saharan Africa

## Abstract

Universal access to reproductive health services entails strengthening health systems, but requires significant resource commitments as well as efficient and effective use of those resources. A number of international organizations and governments in developing countries are putting efforts into tracking the flow of health resources in order to inform resource mobilization and allocation, strategic planning, priority setting, advocacy and general policy making. The UNFPA/NIDI-led Resource Flows Project ("The UNFPA/NIDI RF Project") has conducted annual surveys since 1997 to monitor progress achieved by developing countries in implementing reproductive health financial targets. This commentary summarizes the Project experiences and challenges in gathering data on allocation of resources for reproductive health at the domestic level in sub-Saharan African countries. One key lesson learnt from the Project experience is the need for strengthening tracking mechanisms in sub-Saharan African countries and making information on reproductive health resources and expenditures available, in particular the private sector resources.

## Background

During the special session of the United Nations General Assembly that took place five years after the International Conference on Population and Development in Cairo, health systems stakeholders set a target of universal access to quality reproductive health services by 2015 [1]. It is acknowledged that universal access to reproductive health and rights for women and girls is a necessary pre-condition for fighting poverty; improving reproductive health is not a specific Millennium Development Goal, but simply fundamental for fighting poverty and attain sustainable development [1,2]. Despite gains made in reproductive health indicators^a^ in sub-Saharan African countries, marked disparities persist across and within countries [2,3]. Universal access to reproductive health services entails the strengthening of health systems, which requires significant resource commitments as well as efficient and effective use of those resources. Recent international meetings such as the international family planning conferences in Dakar (2011) and Addis Ababa (2013) and the family planning summit in London (2012) united donor and African countries’ leaders around new funding commitments to expand access to family planning methods [4].

In order to properly address funding issues, country leaders first need to understand how much is currently available and being spent on reproductive health, what activities are being paid for, and where adjustments need to be made. Often, governments in sub-Saharan Africa do not have the critical information they need to plan budgets that would allow them to achieve their reproductive health goals [5,6]. Civil society also lacks information about where money is going, and is thus unable to lobby successfully for national and international funds to fill the gaps. The challenge is, therefore, to obtain information that will lead to more effective use of the resources available.

Since 1997, the UNFPA/NIDI-led Resource Flows Project ("The UNFPA/NIDI RF Project") has conducted annual surveys to monitor progress achieved by donors and developing countries in implementing the financial resource targets agreed upon at the International Conference on Population and Development in Cairo in 1994 and the Declaration of Commitment adopted at The United Nations Special Session on HIV/AIDS in 2001. The UNFPA RF Project also endeavored to strengthen the institutionalization of country-owned systems to collect data and produce periodic reports that compare the need for reproductive health funding at the national level with the allocation of resources (domestic and external), actual expenditure and distribution of resources, as well as projected availability of resources (domestic and external) in the years ahead.

The RF project's primary instrument to monitor global financial flows is the annual mail survey. In addition to the mail survey, 15 country case studies were conducted between 1997 and 2002. These studies helped to institutionalize domestic data collection and acquire a better understanding of national systems of financing population and AIDS activities. Since its inception, the RF project has evolved according to the needs of UNFPA and to the challenges faced in collecting accurate, reliable and timely data. In response to needs for more timely data, the RF project produces estimates and projections of resource flows to population and AIDS activities since 2003. Data collection is also strengthened by thematic studies on the use of reproductive health accounts, the allocation of population and AIDS funds in Sector-Wide Approaches (SWAps), and the estimation of private sector expenditures, including household expenses, and lower administrative level expenditures^b^.

The project uses the term “population activities” to refer to projects, programmes and activities in the following categories: a) Family planning services; b) Basic reproductive/maternal health services; and c) Basic research, data and population and development policy analysis. At the core of the RF project’s activities are the donor and domestic surveys, whereby financial data on population and AIDS expenditures are collected at the project/programme level, covering expenditures during the previous year and expected expenditures during the current year and several years ahead.

Data collection for donors started in 1996 and the sample for the 2010 survey included 125 organizations, among which were 24 DAC donor countries, 9 UN agencies, 28 foundations, 58 NGOs, 3 universities and research institutes, and 3 development banks. Data on population assistance are gathered with the use of a detailed questionnaire sent to major players in the field of population and AIDS, whose funding accounts for the majority of funding for population assistance. These include donor countries that are part of the OECD/DAC and the European Union, multilateral organizations and agencies, major private foundations and other international NGOs and development banks that provide substantial population assistance. Most information for donor countries is obtained from the OECD/DAC database.

Data collection for the Domestic Survey started in 1996 and since 2008 the sample includes 171 developing countries and countries in transition. Data on domestic resources are collected via an annual survey sent by e-mail to UNFPA Country Offices for further distribution to government departments and national NGOs. A separate questionnaire for national consultants asks for information on the national budget, future commitments and the private sector. In addition, the consultant is requested to write a report on the data collection process, which provides more information on coverage, quality of data, and problems encountered during follow-up and response. Data collected are based on: 1) primary sources; 2) actual expenditures (not commitments); 3) restricted to public sector (government and NGOs); and 4) include project level information to avoid double counting. Questionnaires for governments are for distribution to those departments that are involved in population activities, for example, Ministries of Health, Population, Education, or Central Statistical Offices, government-run research centres or universities. Questionnaires for national NGOs are for distribution to national non-governmental, not-for-profit organizations involved in population activities that are responsible for more than about one percent of the total funds for population activities in the country.

This comment focuses on the challenges associated with gathering data in the domestic survey, mainly issues related to countries’ willingness to participate in the survey, data availability and data sharing.

## Countries’ willingness to participate in the domestic survey

The number of sub-Saharan African countries willing to participate in the study increased over the years, from 16 countries in 2002 to 39 countries in 2013. Burundi and Madagascar had the highest number of participation over the years (10 participations each). The lowest numbers of participations were registered for Mauritius (2005 only), Namibia (2002 and 2011), Sao Tome and Principe (2012 and 2013), Lesotho (2011 and 2013), Equatorial Guinea (2012 and 3013) and Gabon (2013 and 2013). Reasons for non-participation over the years included: human and financial constraints in implementing the data collection process at UNFPA level, data unavailability, and census workload. No responses were registered in 2011 for Comoros, Liberia, Mauritania, Mauritius, Nigeria and Sierra Leone, and in 2012 for Zambia, Namibia, Lesotho, Ghana and Gabon.

## Data availability and data sharing

Apart from response rates, many challenges have affected data completeness and survey timelines over the years. These challenges mainly stemmed from bureaucratic bottlenecks, lack of updated and comprehensive national databases, limited accounting processes in various countries, and political instability. In various countries, delays in data collection were observed due to restrictive in-organization clearance rules. Questionnaires sent to government departments were sometimes kept for months pending clearance from the department heads or permanent secretaries often engaged in other activities outside the country. Cases where the focal person appointed to fill out the questionnaires left without handing them over to the next person were also experienced in some countries. In various countries, the lack of comprehensive and updated national databases has rendered difficult the process of identifying key organizations dealing with population activities.

In terms of data availability, limited accounting processes and poor record keeping remain the key issue when collecting data on health financing in sub-Saharan African countries. Difficulties in estimating budget and expenditures for specific health subcategories (e.g. reproductive health, family planning, HIV/AIDS) or in estimating the private sector expenditure (e.g. household spending, private insurances spending) were encountered in various countries. It was also quite challenging in some of the countries to estimate administrative costs for population activities (e.g. staff emoluments, utilities, transport cost). Table 1 below gives a snapshot of the types of challenges encountered.

**Table 1:** **Challenges faced in collecting data on reproductive health financing in sub-Saharan African countries**

**Country**	**Limitations/Challenges**
**Country – West Africa**	• Underestimation of public expenditures (exemptions and personnel costs are not sufficiently taken into account).
	• Households’ expenditures occupy a significant portion of health expenditures that are not considered by organizations while filling out the questionnaires.
	• Political instability: the period for the data collection coincided with the Burkina political instability. Many employers have been affected by the events of 30 October 2014 and could not be investigated. Some business leaders and NGOs have still not been found due of these events. Moreover, the late transmission of information by certain structures has been a common/persistent issue.
**Country – East Africa**	• Reluctance to provide data, especially by government departments.
	• Delay by NGOs in completing the questionnaires.
**Country- Central Africa**	• Plurality of persons involved in the filling of questionnaires: projects and programs for population activities and/or family planning are usually managed by several people; the person responsible for completing the questionnaires does not always have all the necessary information and is forced to appeal to the leaders of projects and programs, who are in turn often not available.
**Country- Central Africa**	• Reluctance of some NGOs to provide the requested information.
	• Incomplete questionnaires returned by some NGOs.
**Country- Central Africa**	• Unavailability of data in the form requested due to the lack of organization/documentation of statistical information in the various public and private administrations.
	• Budgets are often developed in a comprehensive manner and the disaggregation into specific components of population activities is not easy.
	• Unavailability of technical expertise to help disaggregate data, leading to non-responses.
**Country- Southern Africa**	• Accuracy of data: In the case of government budgets and expenditures, it was difficult to disaggregate and differentiate the components of the ‘costed package’ from all other areas of population and development spending. Estimates had to be provided because relevant population activities are commonly integrated within general development projects, and amounts are given as huge lump sums.
	• Financial reporting systems: Various institutions have different financial reporting systems, which makes it difficult to provide data in the format required by the survey.
	• Non response: Obvious reluctance by some government ministries to provide data mainly because of the challenge regarding categorization of budgets. Furthermore, the fact that the survey required giving definite amounts, including those specific for projects, posed a great challenge. In most institutions, employees responsible for project management of population activities are not well informed of the allocation of funds that go hand-in-hand with their projects. As such, questionnaires required staff from both the accounts and the project/programme departments to work together in providing the information. This alone took more time, and both parties showed lack of commitment in filling in the questionnaire.
**Country- Southern Africa**	• There are no benchmarked funds for various population activities and money disbursed is put in one basket.
	• State budget is not disaggregated by types of activity and this makes estimation of domestic resources for population activities difficult.
**Country- Southern Africa**	• Categorizations of expenditure within health include women and children at the lowest level. Expenditure is also recorded for strategic objectives, of which population activities are equally distributed across the board. Within the strategic objectives, sub-programmes do not have earmarked funds.
	• Current financial tracking data that exists within the health sector is very poor. It is possible to identify the total funding that goes to various provinces and districts, but disaggregating this within the programme area is not possible for maternal and reproductive health. Current accounting systems within the Department of Health classify spending within the broader category of Maternal, Child and Women’s health.
	• The focus of spending has also shifted to more integrated systems, which makes it harder to track individual spending. Spending for activities come from various funding streams. Within hospitals, the hospitals are funded as a whole and individuals spread their time across sectors, hence an analysis of expenditure at health care level was not feasible. In order to accurately determine spending at this level a time-motion study would be required.
	• Population and research expenditure falls across a number of government bodies and the source of these funds, while mostly domestic, are from various streams. Hence, it is difficult to track expenditure.
**Country- Eastern Africa**	• Non-cooperative respondents/institutions even after several call-backs.
	• Inconsistencies in the data provided. Some institutions provided figures for similar reference years with different data.

## Lessons learnt and the way forward

A crucial lesson learnt from the UNFPA/NIDI Resource Flows Project experience is about the need to strengthen sub-Saharan African countries’ awareness of the value of tracking resources for health in general and reproductive health in particular and the value of sharing these data with the research community. As rightfully acknowledged by the World Health Organization and partners, “countries cannot manage what they cannot measure”. The World Health Organization has provided developing countries with valuable tools and technical support to institutionalize and set up harmonized platforms for timely collection of health expenditure data over the last 20 years. With the support from the World Health Organization and other major international organizations, national health account (NHA) has been conducted in 22 countries in sub-Saharan Africa. In addition to a national account of all health-related expenditures, subaccount analyses were conducted in some countries for a number of specific health areas, such as Human Immunodeficiency Virus/Acquired Immune Deficiency Syndrome (HIV/AIDS), Reproductive Health (RH), tuberculosis, malaria, and child health. These subaccounts provide detailed assessments of the flow of funding for priority health areas. Reproductive health account subaccounts estimate total spending on FP and maternal health. National health accounts constitute the main accounting framework in most sub-Saharan African countries, but its institutionalization in countries’ public expenditure management systems remains a challenge. The following are some key steps that can be taken in order to institutionalize the national health account process in sub-Saharan African countries and increase countries’ capacities to undertake such monitoring activities:

- Expand the NHA team to include the representation of key NGOs and development partners. This will ideally ensure that these key players understand and appreciate what kind of information the RHA accounts require and why.
- Development of a standardized data collection tool that targets the donors and NGOs. This can later be computerized so that expenditure information on reproductive health, for instance, is made available on a regular basis from the NGOs. This should be followed by an intensive advocacy to ensure that the tool be utilised on regular basis and the data then sent to the NHA team. A more targeted training in universities to create a critical mass of NHA practitioners.

Another important lesson learnt from the Resource Flows Project experience is the need to advocate for collecting refined data to track and monitor progress in implementing financial commitments of sub-Saharan African countries towards key reproductive health goals such as the FP2020 goal. Initiated in the wake of the 2012 London Summit on Family Planning, the FP 2020 initiative aims at accelerating the momentum and building the foundation of the global movement to reach 120 million more women and girls worldwide with access to voluntary family planning by 2020. Gathering specific information from governments, corporations, insurance companies, NGOs and local philanthropies on income received or generated, expenditures, payments or reimbursements for family planning commodities and services on a yearly basis will provide valuable monitoring input in order to track countries’ commitments and achievements in reaching the highest possible numbers of women and girls with access to voluntary family planning by 2020. Our experience with the Resource Flows Project indicates that many countries in sub-Saharan Africa encounter challenges in providing disaggregated health data where funds used for reproductive health and family planning activities are clearly identified. This can be explained by the fact that very few countries comply with the need to generate reproductive health sub-accounts [6]. Countries’ willingness to track and share refined information on reproductive health and family planning expenditures will be key in determining the quality of data gathered by the Resources Flows Project or any other similar type of data collection.

### Endnotes

^a^Such as gains in addressing unmet needs for family planning, increasing contraceptive prevalence, and preventing unwanted pregnancies.

^b^For further information about the project and data collection see the resource flows website, http://www.resourceflows.org.

## Abbreviations

UNFPA: United Nations Population Fund
NIDI: Netherlands Interdisciplinary Demographic
